# Excellent survivorship and improved active shoulder range of motion 5 years post-reverse total shoulder arthroplasty with mini-augmented glenoid baseplate and mini-humeral tray

**DOI:** 10.1016/j.jsea.2026.100103

**Published:** 2026-09-30

**Authors:** Ryan Krupp, Christopher O'Grady, Brian Werner, J. Michael Wiater, John Nyland, Thomas Duquin

**Affiliations:** a Louisville Institute of Orthopaedics, Louisville, KY, USA; b Andrews Research and Education Foundation, Gulf Breeze, FL, USA; c Department of Orthopaedic Surgery, University of Virginia, Charlottesville, VA, USA; d Department of Orthopaedic Surgery, Beaumont Health, Royal Oak, MI, USA; e Department of Orthopaedic Surgery, University of Louisville, Louisville, KY, USA; f Department of Orthopaedics, State University of New York at Buffalo, Buffalo, NY, USA

**Keywords:** Patient outcome, Shoulder function, Quality of life, Glenoid, Scapular notching, Range of motion

## Abstract

**Background:**

Following reverse total shoulder arthroplasty (rTSA) scapular notching and biomechanical impingement can increase pain and decrease function. Mini–augmented glenoid baseplate (AGB) and offset humeral tray use may improve biomechanics, correct glenoid bone deficiency, and decrease complications. This 5-year post-rTSA study is the follow-up to a previous 2-year patient outcome study. The hypothesis was that 5-year patient outcomes would be comparable or better than those observed at 2 years with the lower implant profile group displaying superior active range of motion (ROM) and patient-reported outcome measurement (PROM) results.

**Methods:**

Ninety-eight patients received 1 of 3 different glenoid baseplate and humeral tray combinations: group 1 (mini-AGB and a 44-mm concentric humeral tray), group 2 (non-AGB and a 40-mm, mini-humeral tray with 3 offset options), or group 3 (mini-AGB and 40-mm mini-humeral tray with 3 offset options). Patients underwent radiologic evaluations, completed the American Shoulder and Elbow Society shoulder score, the EuroQol 5-Dimension 5-Level questionnaire quality of life scale, visual analog scale shoulder pain and instability questions, and underwent active shoulder ROM measurements pre-rTSA, and 6 weeks, 6 months, 1, 2, 3, and 5 years post-rTSA.

**Results:**

Fifty-eight subjects completed 5-year follow-up. More group 2 subjects had mild glenoid erosion and more group 1 subjects had moderate glenoid erosion (Chi-square = 14.6, *P* = .006). More group 3 subjects had subscapularis repair (Chi-square statistic = 6.8, *P* = .03). Across the 5-year post-rTSA study period, subjects who received the combined mini-AGB and mini-humeral tray with offset option (group 3) had greater overall active shoulder flexion than group 1 (*P* ≤ .04), greater active shoulder external rotation (90° abducted) than group 2 (*P* ≤ .05), and greater active shoulder internal rotation than groups 1 and 2 (*P* < .001). Effect sizes were small (0.23 – 0.24). Excellent implant survival (98.3%), minimal scapular notching, and comparable PROM were observed in each group.

**Conclusion:**

Greater active shoulder ROM was observed in subjects that received the lower profile mini-AGB and mini-humeral tray. High implant survival, low scapular notching rates, and PROM improvements were observed for all groups.

Advanced osteoarthritis (OA) may alter glenohumeral joint biomechanics, accelerating rotator cuff degeneration.^48^ Glenohumeral joint OA, rheumatoid arthritis, periarticular fractures, chronic instability, tumors, and glenoid bone loss can each increase shoulder arthroplasty difficulty.^27^ Primary anatomic total shoulder arthroplasty (aTSA) patient outcome success is greatly reduced in the presence of functional rotator cuff deficiency and glenoid bone loss.^14^, ^15^, ^32^, ^43^ Insufficient glenoid bone stock may contribute to shoulder arthroplasty implant loosening, decreasing survival.^18^, ^44^ When aTSA cannot achieve stable fixation, reverse total shoulder arthroplasty (rTSA) may be effective.^4^, ^12^, ^24^, ^37^ Glenoid deterioration, however, may similarly jeopardize rTSA glenoid baseplate alignment and fixation, increasing scapular notching, impairing kinematics and soft tissue tension, and increasing instability or impingement.^19^, ^20^, ^21^, ^28^, ^36^, ^49^, ^51^

Up to 90% of rTSA patient cases may present with scapular notching,^29^, ^30^, ^59^ and radiographic evidence can occur as early as six weeks post-rTSA.^18^ Scapular notching generally starts as a biomechanical process eventually creating polyethylene wear, debris accumulation, and biological osteolysis.^54^, ^55^, ^56^, ^59^, ^63^ By 5 years post-rTSA, scapular notching may be associated with decreased function, reduced mobility, impaired strength, and higher revision rates.^16^, ^26^, ^42^, ^52^ Bone grafting has been historically used to manage glenoid bone loss, but highly variable patient outcomes have been reported with these technically demanding surgical approaches,^24^, ^32^ particularly when attempting glenosphere lateralization.^32^, ^44^ Without glenoid deficiency correction, patients are at greater rTSA implant impingement, loosening, and earlier revision risk.^32^, ^44^ With increasing rTSA use in patients with wider age ranges and higher function expectations, identifying a glenoid deficiency solution that decreases scapular notching is important.^24^, ^42^ Humeral neck-shaft angle, center of rotation offset, glenosphere diameter and tilt, implant size, congruency, and fit may each influence rTSA fixation and bony integration, shoulder mobility, and implant survival.^2^, ^13^, ^21^, ^33^, ^44^, ^52^ This study is the 5-year follow-up of a previously reported 2-year outcome study of patients post-rTSA using 1 of 3 different glenoid baseplate and humeral tray combinations from the same manufacturer.^31^ To enhance rTSA function, modified implant designs combining improved glenoid fit/congruency through lower profile components may help lateralize the center of rotation, increasing impingement-free motion arcs. The study hypothesis was that 5-year patient outcomes would be comparable or better than those observed at the 2-year follow-up and that the group receiving the lower profile mini–augmented glenoid baseplate (mini-AGB) and 40-mm mini-humeral tray (with 3 offset options) combination would display superior active range of motion (ROM; primary outcome) and patient-reported outcome measurement (PROM; secondary outcome) results.

## Materials and methods

### Study design

This was a retrospective study of prospectively collected data that included patients treated by 6 fellowship trained shoulder surgeon co-investigators, each from different clinical research sites. Medical institutional review board study approval was sought and obtained from each site (CMG2017-70E, Western Institutional Review Board, Inc., Puyallup, WA, USA) and all study subjects provided written informed consent. Previous study^31^ and pilot testing suggested that a 10° mean active shoulder ROM difference (primary outcome) between high and low profile rTSA implant groups with a 10° common standard deviation with 8 repeated measures required at least 11 subjects/group for a statistical power of 80% at an alpha value of *P* ≤ .05.

### Inclusion criteria

To be considered for study inclusion, patients had to be ≥ 18 years of age, have rotator cuff deficiency with arthropathy, a rotator cuff tear with OA, rheumatoid arthritis, proximal humerus fracture, or a massive rotator cuff tendon tear. Patients also had to have a functioning deltoid muscle and be anatomically and structurally suited for rTSA.

### Exclusion criteria

Patients were excluded from study participation if they had a neurologic disorder (nerve injury, brachial plexopathy, paralytic/non-functional deltoid muscle), shoulder osteoporosis, or osteomalacia (diagnosed either via DEXA scan by a patient's general health physician or by study surgeon pre-rTSA radiograph review); if they had a metabolic disorder that could impair bone formation thereby preventing implant placement; had rapid glenohumeral joint deterioration; or if marked bone loss or resorption was observed on radiographs. Patients were also excluded if they had undergone a failed primary aTSA or rTSA, if they had a distant infection foci that could spread to implant sites, or if they were incapable or unwilling to adhere to study rehabilitation and follow-up protocols.

### Subject selection and regulatory control

Subjects were enrolled between September 2018 and December 2019. Ninety-eight consecutive patients with a primary diagnosis of shoulder OA, rheumatoid arthritis, post-traumatic arthritis, rotator cuff, or other-related conditions that met the inclusion criteria entered the study prior to rTSA with a goal of maintaining high continued subject participation for future 10-year final study completion. Most subjects (72%, 42/58) were referred by the same 2 surgeons (group 1 = 92.3%, group 2 = 94.5%), while 96% of group 3 subjects were recruited by 4 study surgeons. Subjects were assigned to rTSA study groups based solely on the surgeon's pre-operative assessment of functional needs and expectations, shoulder radiographs, and intra-operative findings including glenoid morphology/bone loss severity, bone quality, and history. Each surgeon selected the implant combination and placement that they believed would provide the best possible patient outcome. Augmented glenoid components were used not only in cases with glenoid bone loss/deformity but also to achieve optimal glenoid implant alignment and fixation while minimizing reaming and preserving bone. The mini-humeral tray used in groups 2 and 3, however, was not available when group 1 subjects were assigned. Group 1 subject cohort data were collected first, using the rTSA components available during that time period. Surgeries took place between October 2018 and December 2020. The study sponsor regulatory team provided surgical and follow-up evaluation protocol adherence oversight through clinical research study coordinators and offered additional assistance as needed.

### Reverse total shoulder arthroplasty component group assignment

Subjects were assigned to rTSA groups based on the surgeon's pre-operative assessment of their functional needs and expectations, shoulder radiographs, and intra-operative findings including glenoid morphology/bone loss severity, bone quality, and history. Study surgeons selected the implant combination and placement they believed would provide optimal patient outcomes. Augmented glenoid components were used not only in cases with glenoid bone loss/deformity but also to achieve optimal rTSA implant alignment while minimizing reaming and preserving bone.

Each subject received 1 of 3 different rTSA implant combinations from the same manufacturer (Zimmer Biomet, Warsaw, IN, USA) with at least 1 design difference. At the humerus, subjects received either a standard humeral tray (44-mm diameter) with a porous tantalum-coated proximal titanium humeral stem and central trunnion, or a mini-humeral tray (40-mm diameter) with 3 trunnion offset options. At the glenoid, subjects received either a mini-glenoid baseplate, or a mini-AGB (Fig. 1, *A-C*). Subjects completing each follow-up visit over the 5-year study period included the following: group 1 (n = 13), mini-AGB, and standard humeral tray (44-mm diameter) with central trunnion; group 2 (n = 18), standard glenoid baseplate, and mini-humeral tray (40-mm diameter) with 3 trunnion options; and group 3 (n = 27) both a mini-AGB and a mini-humeral tray (40-mm diameter) with 3 trunnion options.

**Figure 1:**
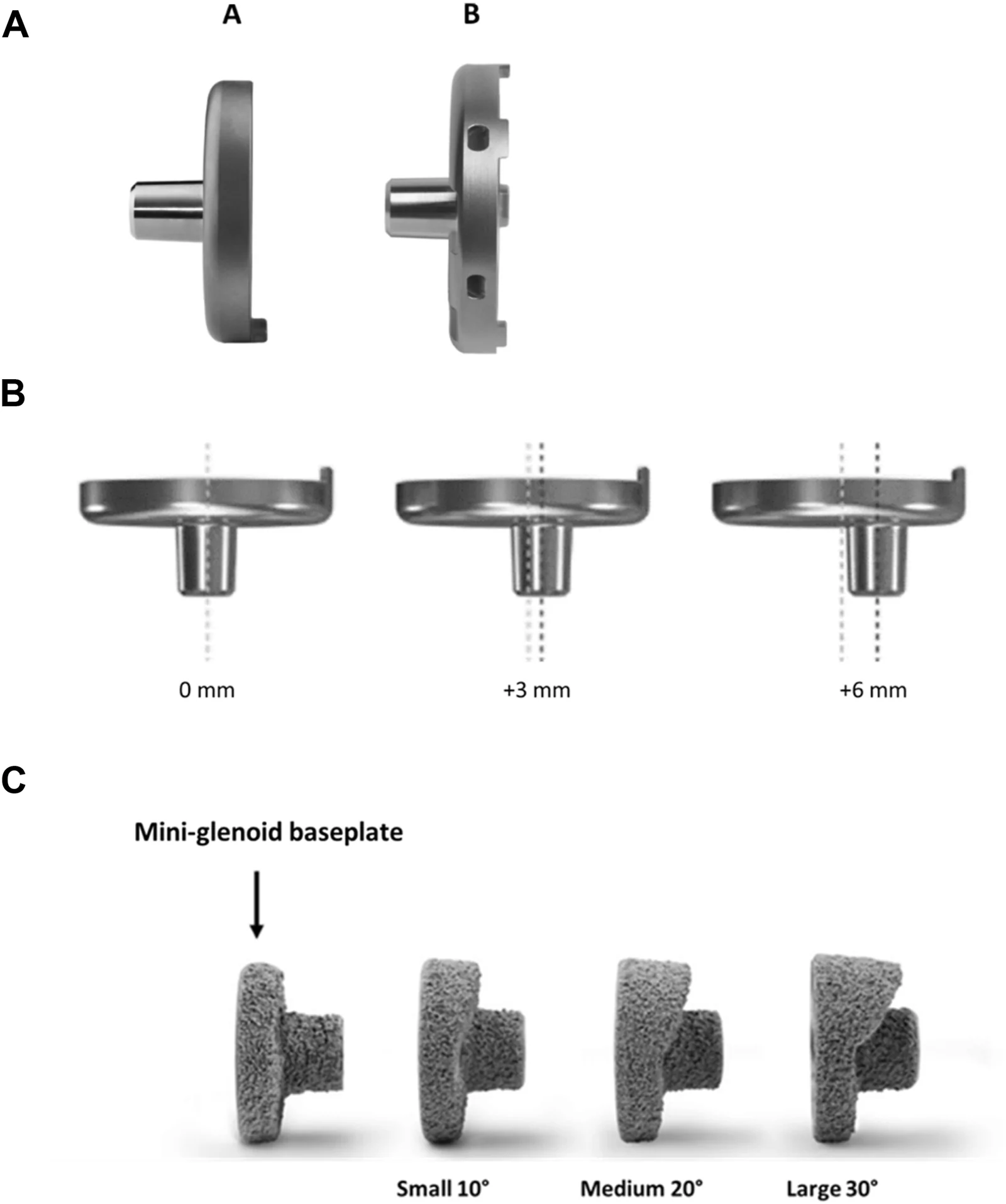
(**A**) Mini humeral tray (40 mm) (**A**) and standard humeral tray (44 mm) (**B**). (**B**) Humeral tray trunnion offset options (0 mm, +3 mm, +6 mm). (**C**) Standard mini-glenoid baseplate augmentation options.

### Operative technique

Study shoulder surgeons followed the same previously reported surgical technique.^62^ Patients were in the upright beach chair position during surgery with their head firmly secured and the contralateral arm either draped free, or placed in an arm holder. To enable restricted shoulder adduction and hyperextension, the operative arm was positioned off the side of a surgical table. All patients received an interscalene nerve block in addition to general anesthesia. A standard deltopectoral surgical approach was used.

### Rehabilitation protocol

Subjects followed the same basic rehabilitation guidelines,^8^ but slight progression timing differences may have occurred between clinical research study sites. Any progression timing difference that may have occurred, however, was not based on rTSA implant design modifications. In general, rehabilitation required sling use and non-weightbearing upper extremity status over the initial 4 weeks post-rTSA. In a controlled setting, daily time out of the sling was encouraged. A four-phase rehabilitation plan was followed. *Phase I (post-rTSA weeks 1–4)*: supported pendulum exercises and isometric deltoid muscle activation, and gentle elbow, wrist, and hand active mobility (squeezing a ball) were encouraged. In supine, active shoulder, external rotation to 20° and forward elevation to 90° was allowed. During this phase, shoulder internal rotation past waist level was not allowed. *Phase II (post-rTSA weeks 5–6)*: during this phase, active shoulder external rotation progressively increased, full forward shoulder elevation within pain-free limits progressed from supine to sitting, and shoulder internal rotation gradually increased to full ROM. *Phase III (post-rTSA week 7–12)*: after pendulum exercises, upper extremity proprioceptive neuro-muscular facilitation with scapular integration was performed, and progressive resistance external and internal shoulder rotation, seated rows, shoulder shrugs, biceps curls, and “bear hug” exercises were initiated. *Phase IV (≥12 weeks post-rTSA)*: progressive resistance weight training within pain-free limits continued with reminders to keep “hands within eyesight,” maintain slight elbow flexion, and minimize overhead movements. Military press, behind the head latissimus dorsi pulldowns, and wide grip bench presses were contraindicated. Subjects were generally allowed to return to computer work at 4 weeks, golf at 3–4 months, and tennis by 4–6 months post-rTSA as desired.^31^

### Study measurements

Pre-operatively and at 6-week, 6-month, 1-year, 2-year, 3-year, and 5-year post-rTSA follow-up visits, subjects completed the American Shoulder and Elbow Society (ASES) shoulder score, the EuroQol 5-Dimension 5-Level questionnaire (EQ-5D-5L; 5-component, 5-level quality of life scale assessing mobility, self-care, usual activities, pain/discomfort, and anxiety/depression), and visual analog scale (VAS) shoulder pain and instability scores. Active shoulder ROM and perceived functional strength were also evaluated.

With the exception of shoulder internal rotation, a handheld goniometer was used to measure active shoulder ROM.^46^ Shoulder internal rotation was assessed by having the patient actively place the dorsum of their hand behind their back and slide it up as far as possible from the coccyx to an upper thoracic vertebrae spinous process. The highest level that could be reached with the thumb tip was recorded as maximal shoulder internal rotation. The coccyx was assigned a score of level 1, and each more proximal spinous process represented an additional level, culminating at level 18 for thoracic spinous process #4. Similar methods using a flexible tape measure reported moderate-to-good inter-rater reliability.^39^ Pilot testing of 10 subjects and 3 raters using this method revealed similar good measurement reliability (intraclass correlation coefficient = 0.89). Perceived shoulder strength was estimated using a Likert scale type question regarding subject perceptions of their ability to lift a 10 lb. (4.5 kg) object above shoulder level (1 = unable, 2 = very difficult, 3 = somewhat difficult, 4 = not difficult).

### Radiographic imaging assessments

True anteroposterior (Grashey) and axillary radiographic images were taken pre-rTSA and at each clinical follow-up visit. Pre-rTSA, computed tomography (CT) scans were also requested based on surgeon preference, perceived pre-operative radiograph quality, and patient insurance coverage. When available, CT scans were used for pre-operative Walch and Sirveaux classification determination.^6^, ^25^, ^58^ When pre-operative CT scans were not obtained, Walch classifications were determined during intra-operative assessments. The number of subjects with pre-operative CT scans was similar between groups. Glenoid fossa erosion grades were also estimated based on pre-operative imaging and intra-operative shoulder surgeon assessments.^1^, ^49^ At each clinical follow-up visit, the study surgeon reviewed radiographic images to assess glenoid process baseplate or humeral stem subsidence and migration, bony changes associated with implant loosening, and scapular notching.^54^ Scapular notching was graded according to the Nerot-Sirveaux Classification criteria.^61^

### Statistical analysis methods

Kolmogorov–Smirnov and Shapiro–Wilk tests revealed that outcome active ROM values and PROM scores did not always display normal distributions at each measurement interval; therefore, non-parametric statistical analysis was performed. Group continuous score differences were determined using Friedman two-way analysis of variance and Wilcoxon test pairwise comparisons. Group categorical score differences were determined using Chi Square or Fisher exact tests. Group active shoulder ROM change effect sizes (r) were calculated using the standardized pairwise test statistic divided by the square root of the total number of pairwise comparisons, and 95% confidence intervals (CI) were calculated using the following formula (95% CI = effect size ± 1.96 (standard error).^10^ All statistical analyses and graphing were performed using Statistical Package for the Social Sciences software (version 29.0; IBM-SPSS, Armonk, NY, USA). An alpha level of *P* ≤ .05 was selected to indicate statistically significant group differences.

## Results

Subject demographics are reported in Table I. From the initial group of 98 subjects that qualified for study inclusion, a similar proportion from each group completed 2-year (n = 67) and 5-year (n = 58) follow-up (Chi-square statistic = 5.4, *P* = .07) (Fig. 2). Groups had comparable primary shoulder pathology diagnosis frequencies. With the exception of group 2 having more subjects with a history of genitourinary conditions than group 1 and group 3 (Chi-square statistic = 6.9, *P* = .03), groups had similar comorbidity frequencies (*P* > .05). At study participation, however, these comorbidities had either been medically controlled or surgically managed. Pre-rTSA and post-rTSA evaluation and implant information are displayed in Table II. Groups were comparable with the exception of group 2 having more subjects with mild glenoid erosion and group 1 having more subjects with moderate glenoid erosion (Chi-square statistic = 14.6, *P* = .006). Additionally, group 3 had more subjects who underwent subscapularis repair (48%, 13/27), and group 1 had more subjects that did not undergo subscapularis repair (92%, 12/13) (Chi-square statistic = 6.8, *P* = .03. Groups had comparable EQ-5D-5 L quality of life, ASES shoulder function, VAS shoulder pain and VAS shoulder instability scores (Table III).

**Table I:** Groups displayed similar pre-rTSA demographics.

Demographic characteristics	Group 1 (n = 13)	Group 2 (n = 18)	Group 3 (n = 27)	*P* value
Age (years)	69.4 ± 7	67.4 ± 7	71.7 ± 8	.16
Final follow-up (days)	1855 ± 98	1845 ± 69	1867 ± 63	.61
Shoulder symptom duration (months)	28.7 ± 25	29.6 ± 56	47.8 ± 58	.35
Sex (male, female)	6,7	7,11	14,13	.69
BMI (kg/m^2^)	31.0 ± 6.1	29.8 ± 3.2	30.6 ± 6.8	.85
Tobacco use (yes, no)	0,13	2,16	0,27	.10
Alcohol use (yes, no)	7,6	6,12	14,13	.40

*BMI*, body mass index.

**Figure 2:**
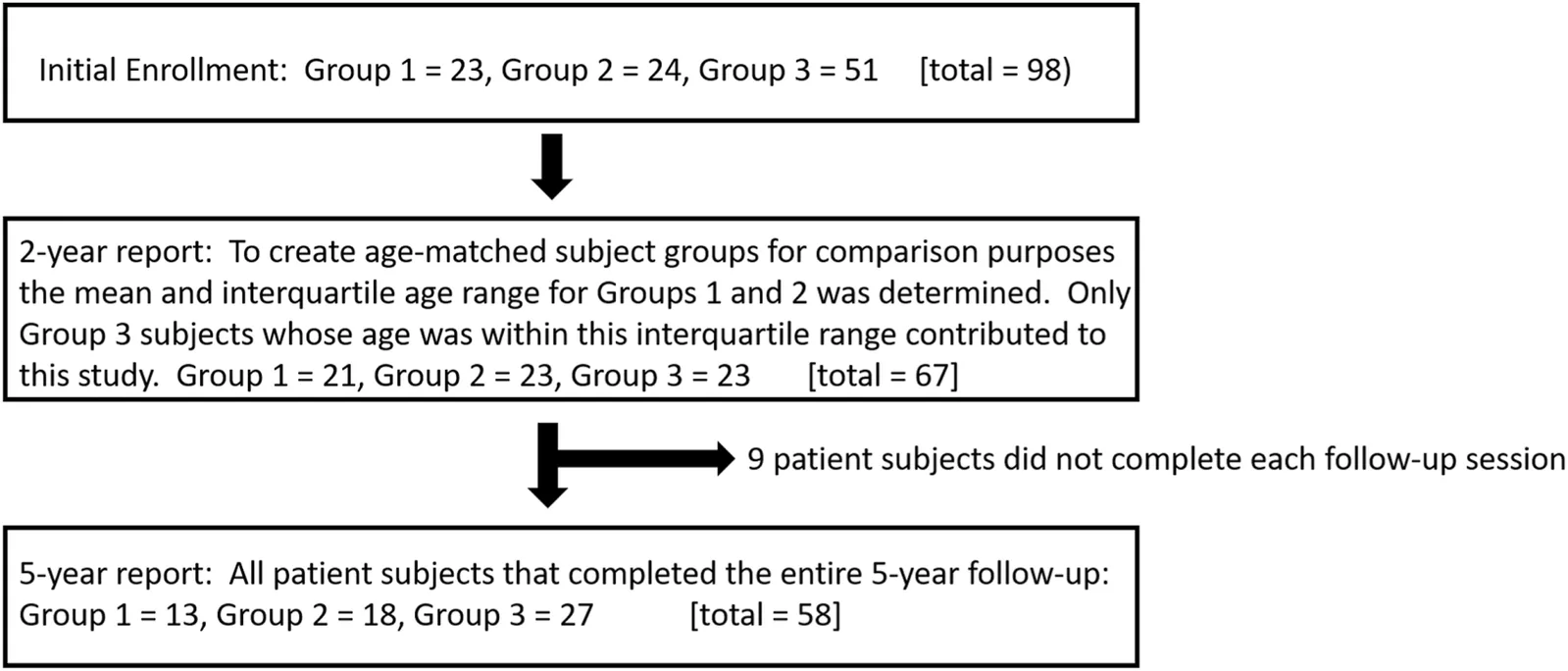
Study subject allocation.

**Table II:** Groups displayed comparable pre-surgical and surgical characteristics and rTSA device-related information with the exception of group 2 having more patients with mild glenoid erosion than the other groups.

Pre-surgical evaluation	Group 1 (n = 13)	Group 2 (n = 18)	Group 3 (n = 27)	*P* value
Primary diagnosis	Rotator cuff arthropathy (RCA) = 9 Shoulder osteoarthritis (OA) = 1 Other = 3	RCA = 11 Shoulder OA = 4 Other = 3	RCA = 10 Shoulder OA = 10 Other = 7	.19
Dominant shoulder (right, left)	12,1	16,2	25,2	.90
Surgical shoulder (right, left)	6,7	10,8	17,10	.77
Sivareaux class (no pre-surgical CT, 0, 1, 2, 3, 4)	7,0,3,1,2,0	12,3,1,1,1,0	15,1,3,6,1,1	.26
Walsh class (no pre-surgical CT, A1, A2, B1, B2, B3, C1, C2)	7,0,2,1,2,1,0	12,5,0,0,0,1,0	15,4,1,1,2,3,1	.30

Surgical evaluation	Group 1 (n = 13)	Group 2 (n = 18)	Group 3 (n = 27)	*P* value
Scapularis tendon management	Not repaired = 12 *(did not want = 8, unable = 4)*; repaired = 1	Not repaired = 13 *(did not want = 7, unable = 6);* repaired = 5	Not repaired = 14 *(did not want = 5, unable = 9);* repaired = 13	.03∗
Walsh wear pattern observed at surgery (not clear, A1, A2, B1, B2, B3, C1, C2)	1, 2, 3, 1, 3, 3, 0, 0	1, 9, 6, 1, 0, 1, 0, 0	1, 10, 3, 5, 4, 2, 1, 1	.26
Glenoid erosion (mild, moderate, severe)	3, 8, 2 *(greater % moderate)*	15, 2, 1 (*greater % mild)*	14, 9, 4	.008∗

Surgical implant information	Group 1 (n = 13)	Group 2 (n = 18)	Group 3 (n = 27)	*P* value
Glenoid augment direction	Superior-posterior = 7; Superior = 3; posterior = 2; anterior = 1	NA	Superior-posterior = 11; superior = 13; posterior-inferior = 2, posterior = 1	.31 (groups 1 and 3 did not differ)
Glenoid baseplate size	Mini-augmented baseplate: small = 8; medium = 4; large = 1	All received standard glenoid baseplate	Mini-augmented baseplate: small = 20; medium = 3; large = 4	.29 (groups 1 and 3 did not differ)
Glenosphere diameter	36 mm = 12; 40 mm = 1	36 mm = 16; 40 mm = 2	36 mm = 23; 40 mm = 4	.25
Glenosphere offset	0 mm = 5; 3 mm = 8; 6 mm = 0	0 mm = 7; 3 mm = 10; 6 mm = 1	0 mm = 23; 3 mm = 4	.11
Humeral tray size	All received a standard humeral tray (44 mm)	All received a mini-humeral tray (40 mm) with 3 trunnion options	All received a mini-humeral tray (40 mm) with 3 trunnion options	Groups 2 and 3 were identical
Humeral tray offset	All received standard humeral tray with central trunnion	3 mm = 6; 6 mm = 11; 0 mm = 1	6 mm = 11; 3 mm = 4; 0 mm = 12	.15 (no group 2 or 3 difference)

*CT*, computed tomography.

∗ *P* < .05.

**Table III:** Groups displayed comparable quality of life, perceived shoulder function, pain, and instability across the 5-y study follow-up (mean ± standard deviation).

PROM	Group	Pre-rTSA	6 weeks	6 mo	1 y	2 y	3 y	5 y	Group	Pairwise comparisons	Effect size (d)
EQ-5D-5 L score	1	73.1 ± 19	77.7 ± 14	84.7 ± 13	80.5 ± 15	80.5 ± 15	82.1 ± 14	81.4 ± 18	NS	NA	NA
	2	77.6 ± 13	76.6 ± 20	84.4 ± 12	84.9 ± 12	89.3 ± 12	84.1 ± 23	84.2 ± 29			
	3	77.0 ± 15	79.6 ± 18	81.5 ± 17	85.2 ± 11	83.0 ± 12	87.3 ± 16	81.1 ± 29			
ASES score	1	31.8 ± 17	62.8 ± 19	86.0 ± 8	88.3 ± 9	88.8 ± 9	86.0 ± 10	83.3 ± 14	NS	NA	NA
	2	33.6 ± 20	64.2 ± 15	81.5 ± 18	84.6 ± 19	88.6 ± 12	87.5 ± 14	81.2 ± 23			
	3	46.0 ± 21	66.6 ± 20	84.9 ± 17	88.1 ± 15	92.2 ± 9	87.5 ± 16	84.4 ± 18			
VAS pain score	1	6.5 ± 2	1.6 ± 2	0.6 ± 1	0.6 ± 1	0.2 ± 0.3	0.4 ± 1	0.9 ± 2	NS	NA	NA
	2	6.0 ± 3	1.8 ± 2	1.2 ± 2	0.9 ± 2	0.5 ± 1	0.6 ± 1	0.8 ± 2			
	3	5.3 ± 3	0.9 ± 1	0.03 ± 1	0.5 ± 1	0.1 ± 0.2	0.2 ± 0.3	1.0 ± 2			
VAS instability score	1	3.8 ± 3	1.1 ± 2	0.3 ± 1	0.2 ± 0.3	0.4 ± 1	0.4 ± 1	0.8 ± 2	NS	NA	NA
	2	3.3 ± 4	1.2 ± 2	0.6 ± 1	0.5 ± 1	0.4 ± 1	0.5 ± 1	0.8 ± 2			
	3	2.4 ± 3	1.1 ± 2	0.2 ± 1	0.8 ± 2	0.4 ± 1	0.6 ± 2	0.5 ± 2			

*EQ-5D-5L*, EuroQol 5-Dimension 5-Level questionnaire; *ASES*, American Shoulder and Elbow Society; *VAS*, visual analog scale; *NS*, not significant; *NA*, not applicable.

Group active shoulder ROM results are displayed in Table IV. Across the 5-year study period, group 3 and group 2 had greater active shoulder flexion than group 1 (*P* ≤ .04). Overall, active shoulder flexion 5 years, 3 years, 2- years, 1 year, and 6 months post-rTSA were > at 6 weeks and pre-rTSA (*P* < .001) (Fig. 3). Across the 5-year study period, group 3 had greater active shoulder external rotation (90° abducted) than group 2 (*P* ≤ .05). Overall, active shoulder external rotation (90° abducted) at 5 years, 3 years, 2 years, 1 year, and at 6 months post-rTSA was > pre-rTSA (*P* ≤ .001) and at 6 weeks was > pre-rTSA, *P* = .03) (Fig. 4). Active shoulder flexion and external rotation (at 90° abduction) movements exceeded minimal clinically important differences of 12° and 3°, respectively.^45^ Across the 5-year study period, group 3 had greater active shoulder internal rotation than group 2 and group 1 (*P* < .001). Overall, active shoulder internal rotation at 5 years, 3 years, and 2 years was > at 1 year, 6 months, 6 weeks, and pre-rTSA (*P* ≤ .03). Each of these mean active flexion, external rotation at 90° abduction, and internal rotation improvements displayed small-to-medium effect sizes.^10^ Perceived shoulder strength improvement was comparable between groups.

**Table IV:** Group active shoulder range of motion comparisons (mean ± standard deviation). Across the 5 y follow-up, groups 3 and 2 had greater shoulder flexion than group 1, group 3 had greater shoulder external rotation (90° abducted) than group 2, and greater shoulder internal rotation than groups 2 and 1.

Shoulder function	Group	Pre-rTSA	6 weeks	6 mo	1 y	2 y	3 y	5 y	Group	Pairwise comparisons	Effect size (r)
Flexion (°)	1	77 ± 34	92 ± 22	137 ± 19	145 ± 18	139 ± 17	140 ± 16	134 ± 18	3,2 > 1, *P* ≤ .04∗	5 y, 3 y, 2 y, 1 y, 6 mo > 6 weeks and pre-rTSA (*P* < .001)∗	Group 3 > group 1 0.24 [-0.11 to 0.59] Group 2 > group 1 0.23 [-0.12 to 0.58]
	2	89 ± 40	117 ± 35	150 ± 27	142 ± 31	153 ± 22	150 ± 19	148 ± 23			
	3	111 ± 38	119 ± 30	156 ± 17	155 ± 20	160 ± 25	157 ± 18	157 ± 25			
ER (°) (adducted)	1	10 ± 13	24 ± 12	41 ± 11	49 ± 15	40 ± 10	43 ± 13	55 ± 18	NS	NA	NA
	2	26 ± 33	22 ± 19	64 ± 52	40 ± 25	52 ± 31	49 ± 24	47 ± 24			
	3	30 ± 31	37 ± 36	61 ± 35	47 ± 20	50 ± 18	51 ± 22	50 ± 19			
ER (°) (90° abducted)	1	6 ± 15	39 ± 19	62 ± 20	61 ± 18	70 ± 17	66 ± 23	67 ± 20	3 > 2, *P* ≤ .05∗	5 y, 3 y, 2 y, 1 y, 6 mo > pre-rTSA (*P* ≤ .001∗); 6 weeks > Pre-rTSA, *P* = .03∗	Group 3 > group 2 0.23 [-0.10 to 0.56]
	2	18 ± 26	41 ± 29	56 ± 31	58 ± 27	65 ± 18	65 ± 17	61 ± 22			
	3	31 ± 30	37 ± 22	68 ± 20	66 ± 24	65 ± 27	69 ± 23	74 ± 19			
IR (spinous process level)	1	2.5 ± 3	2.2 ± 3	5.5 ± 4	5.5 ± 4	8.8 ± 5	6.4 ± 5	10.5 ± 2	3 > 2,1, *P* < .001∗	5 y,3 y, 2 y > 1 y, 6 mo, 6 weeks, and pre-rTSA (*P* ≤ .03∗)	Group 3 > group 2 0.25 [-0.10 to 0.60] Group 3 > group 1 0.22 [-0.13 to 0.57]
	2	4.3 ± 4	3.2 ± 3	4.9 ± 3	6.0 ± 4	7.4 ± 3	8.3 ± 4	8.1 ± 4			
	3	6.0 ± 4	4.9 ± 3	8.3 ± 4	8.8 ± 4	9.3 ± 5	9.4 ± 5	10.3 ± 5			
Can you lift 10 lbs. (4.5 kg) overhead? (mean)	1	1.3 ± 1	1.3 ± 1	2.8 ± 1	3.3 ± 1	3.2 ± 1	3.1 ± 1	2.9 ± 1	NS	NA	NA
	2	1.2 ± 1	1.3 ± 1	2.8 ± 1	2.8 ± 1	3.1 ± 1	3.3 ± 1	3.0 ± 1			
	3	1.4 ± 1	1.8 ± 1	3.0 ± 1	3.4 ± 1	3.4 ± 1	3.1 ± 1	3.3 ± 1			

*NA*, not applicable, *NS*, not significant, *ER*, external rotation, *IR*, internal rotation.

∗ *P* ≤ .05.

**Figure 3:**
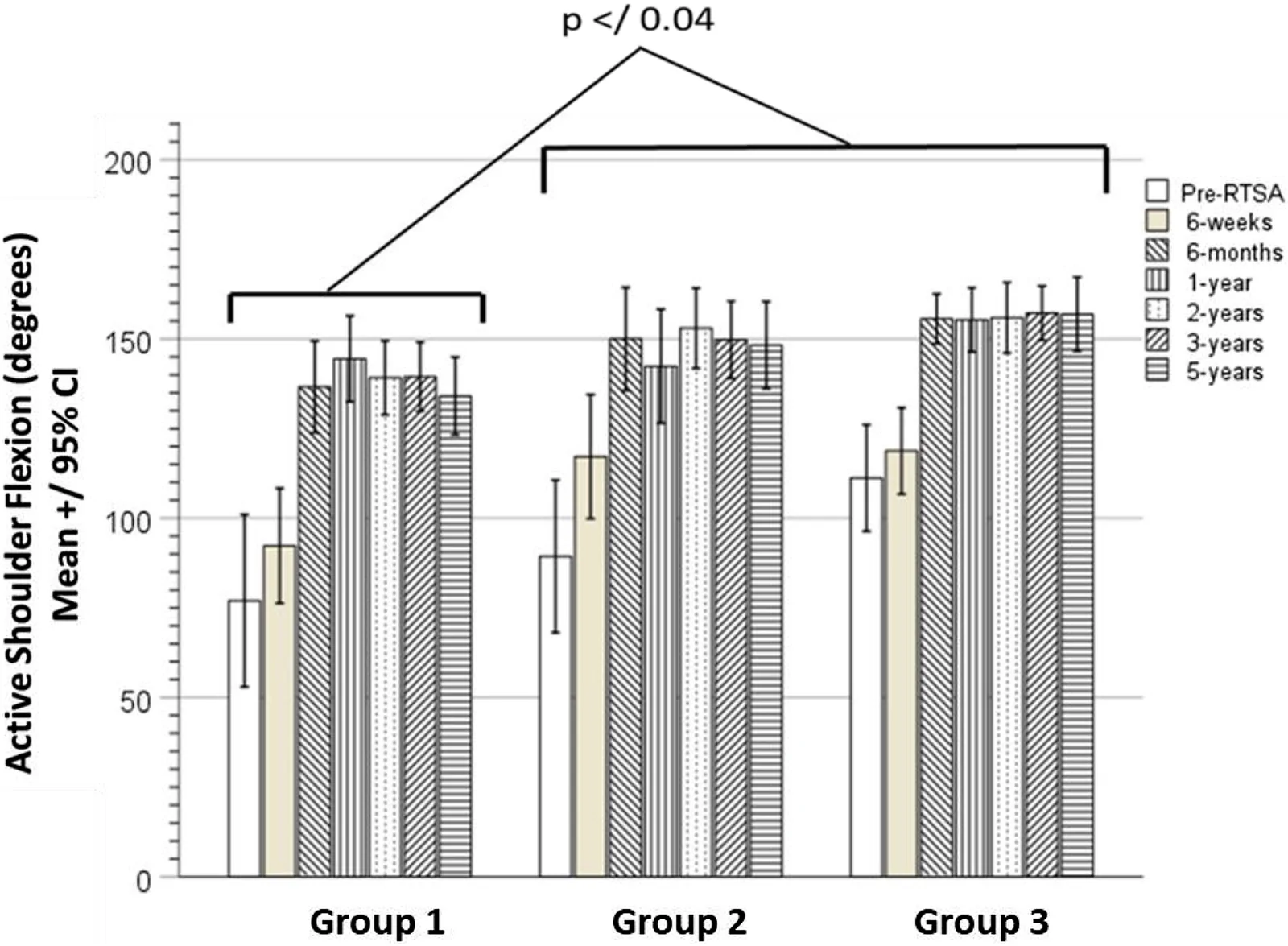
Group 3 and group 2 displayed greater active shoulder flexion than group 1 (*P* ≤ .04) across the 5-year study follow-up. Overall, active shoulder flexion 5 years, 3 years, 2 years, 1 year, and 6 months post-rTSA were > at 6 weeks and pre-rTSA (*P* < .001).

**Figure 4:**
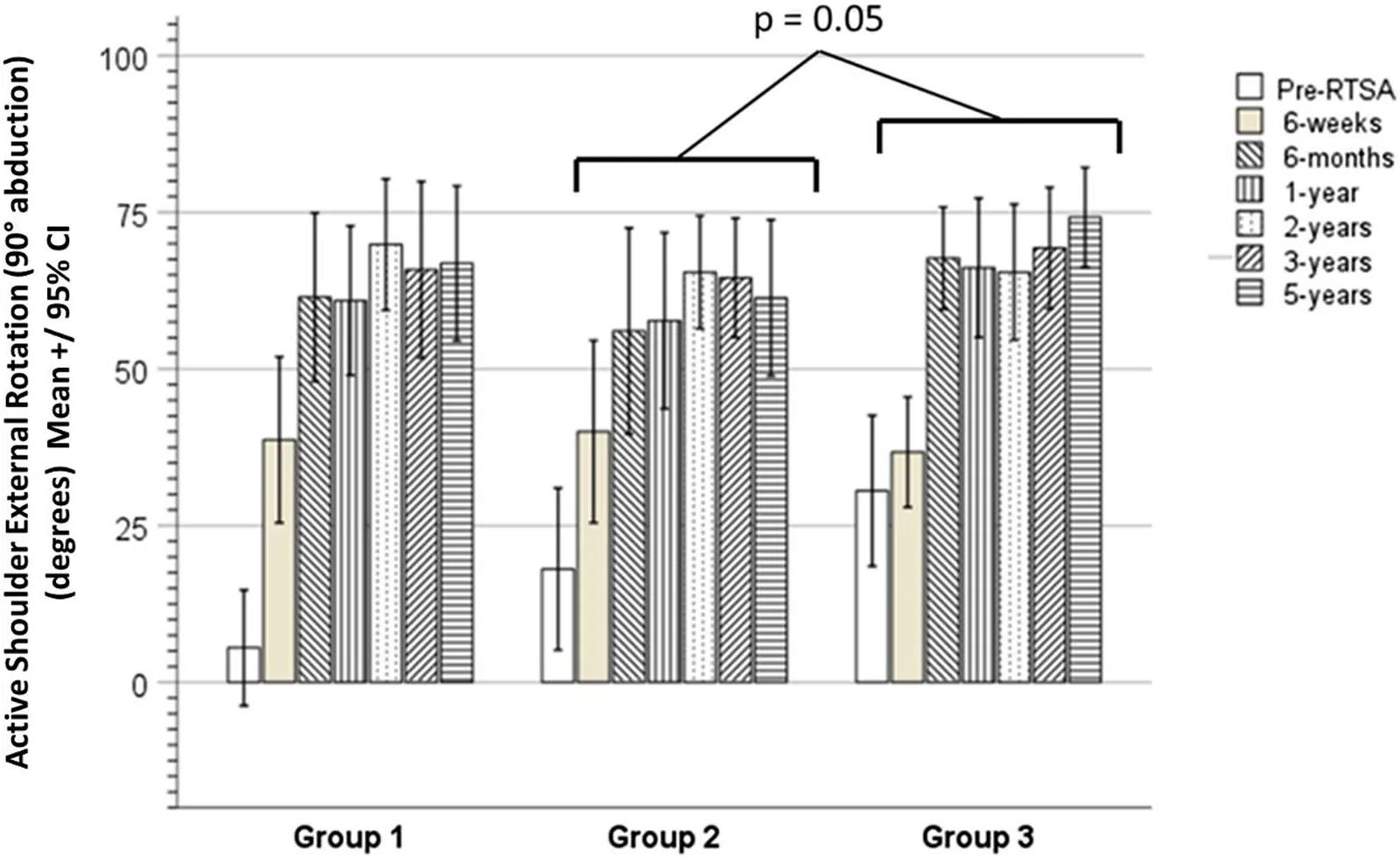
Group 3 displayed greater active shoulder external rotation (90° abducted) than group 2 (*P* ≤ .05) across the 5-year study follow-up. Overall, active shoulder external rotation (90° abducted) at 5 years, 3 years, 2 years, 1 year, and 6 months post-rTSA was > pre-rTSA (*P* ≤ .001) and at 6 weeks was > pre-rTSA, *P* = .03).

Overall, excellent rTSA implant survival was observed (98.3%, 57/58). At the 6 month follow-up, 1 group 3 subject required glenoid baseplate removal with revision due to implant ingrowth failure associated with central screw breakage. Overall, scapular notching incidence was minimal (12%, 7/58) and comparable between groups (*P* = .88). In group 1, 1 subject had grade 2 scapular notching at the 2-year follow-up. In group 2, 1 subject had grade 2 scapular notching at the 3-year follow-up, and 1 subject had grade 1 scapular notching at the 5-year follow-up. In group 3, 1 subject had grade 1 scapular notching at the 2-year follow-up, and 3 subjects had grade 2 notching at the 3-year follow-up. Other significant radiologic findings over the initial 2 years post-rTSA are reported in Table V. Groups did not display significant surgical complication or radiologic finding frequency differences (Chi-square statistic = 0.45, *P* = .80); all complications were conservatively managed and none progressed.

**Table 5:** Groups displayed similar surgical complication or radiologic finding frequency differences across the 5-y follow-up (Chi-square = 0.45, *P* = .80) and all were conservatively managed.

Study group	6 week	6 mo	1 y	2 y	3 y	5 y
Group 1		Implant loosening = 1	Implant loosening = 2			Glenoid component migration = 1
Group 2	Drill bit fragment in glenoid = 1		Calcar rounding, small humeral shaft fracture = 1; heterotopic proximal humeral bone formation = 1		Humeral calcar resorption = 1	Inferior glenoid osteophyte = 1
Group 3			Proximal humeral calcar resorption = 1; proximal humeral calcar rounding and heterotopic bone formation = 1	Osteolysis near triceps tendon origin = 1; humeral shaft osteolysis, small glenoid fracture = 1; humeral shaft osteolysis, heterotopic proximal humeral bone formation, periosteal cortical hypertrophy = 1; humeral shaft osteolysis = 1; humeral osteolysis, periosteal cortical thickening = 1		Glenoid component migration = 1; heterotopic greater tuberosity ossification = 2

## Discussion

Across the 5-year post-rTSA study period, the most important finding was that subjects who received the combined mini-AGB and mini-humeral tray with 3 offset options (group 3) had greater overall active shoulder flexion than group 1 (*P* ≤ .04), greater active shoulder external rotation (90° abducted) than group 2 (*P* ≤ .05), and greater active shoulder internal rotation than groups 1 and 2 (*P* < .001). For all groups, active shoulder ROM was largely restored by 6 months post-rTSA. In contrast, active shoulder internal rotation continued improving through the 2-year post-rTSA follow-up visit. For all groups, the perceived shoulder function and quality of life improvements observed in the 2-year study^31^ were maintained 5 years post-rTSA. By the 5-year follow-up, implant survival was excellent (98.3% survival) and scapular notching was minimal (12%, 7/58) and similar between groups (*P* = .88). The AGB and mini-humeral tray with 3 offset options combination may have enabled more inferior glenosphere placement or more vertical mini-humeral tray alignment, lateralizing rTSA center of rotation, better restoring the physiological deltoid muscle length-tension that contributes to greater active mechanical impingement-free shoulder mobility.^5^, ^7^, ^34^ The greater proportion of subscapularis tendon repair observed in group 3 subjects may also have contributed to this improvement.

Glenoid deficiency in rTSA can make it difficult to achieve fixation, improve stability, and restore shoulder mechanics.^50^ Surgical decisions include incomplete glenoid baseplate seating acceptance, metallic augmentation, and bone grafting.^7^, ^22^, ^38^, ^57^ Whether for primary or revision rTSA, glenoid defect size, shape, and residual bone quality influences the selected surgical method.^35^, ^50^ Metallic augmentation proponents suggest greater “off the shelf” ease of use and more consistent reconstruction longevity compared to bone grafts.^35^ In contrast, fixed “off the shelf” metallic augment implant dimensions may limit their customizability and generalized use.^35^ Because it may better enable precise patient-specific glenoid defect reconstruction, theoretically, autogenous humeral head tissue is an attractive biological glenoid bone stock restoration option.^35^ Bone grafting, however, is technically more difficult to perform and post-surgical bone resorption is concerning.^35^, ^50^ In contrast, metallic implant use is less complicated with no bone resorption risk but with greater cost.^35^

To date, no level I randomized controlled clinical trial has compared rTSA metallic augments to bony augments.^35^ The true impact of successful bone graft healing on patient outcomes has not been fully elucidated since previous studies have evaluated patients with widely varying surgical indications, selection criteria, using differing surgical techniques and bone grafts, with differing radiographic healing assessment methods.^9^ These reasons are likely why bone graft use in primary rTSA glenoid reconstruction has displayed widely ranging (42-100%) patient outcome success rates.^9^, ^50^

Because of deficient bone stock, poor glenoid baseplate fixation increases the likelihood that the rTSA center of rotation will become medialized, leading to insufficient soft tissue tension, instability, and scapular notching.^9^ With independent board-certified radiologist CT scan evaluation, Chamberlain et al^9^ reported that at 2-year follow-up, structural glenoid bone grafting had a 90% complete and 8% partial bony incorporation rate. Significant shoulder pain reduction, improved function, and low revision risk was also observed.^9^ By 4.4 years post-rTSA, Sholtis et al^50^ reported that structural bone autograft or allograft glenoid reconstruction contributed to significant patient ASES score improvements, VAS pain score reductions, and shoulder ROM improvements, exceeding minimal clinically important difference values.^53^ Higher complication (8.5% vs. 3.5%, *P* < .001) and revision (2.4% vs. 0.6%, *P* = .02) rates occurred in the bone graft group than in the augmented metallic implant group. Sholtis et al^50^ concluded that higher bone graft complication and failure rates were associated with its greater use in highly complex, larger volume glenoid bone loss cases, also contributing to reduced post-rTSA shoulder function and clinical outcomes.

In a retrospective study of 447 patients following primary rTSA, Mohamed et al^40^ reported that augmented baseplates performed as well or better than standard baseplates based on ASES, Simple Shoulder Test, University of California, Los Angeles shoulder, Constant shoulder, and Shoulder Pain and Disability Index scores. Significant differences were not observed compared to standard baseplates for complications, revision rates, humeral radiolucent lines, glenoid loosening, or scapular notching.^40^ They concluded that in a severely deformed glenoid, augmented baseplates were a good rTSA option.^40^ In a retrospective study with 22.6 month follow-up, Lo et al^35^ reviewed 3 similar central screw glenoid baseplates with bony or metal augmentation in groups matched for age, sex, and body mass index, reporting no overall glenoid baseplate failure rate differences. Another possible AGB use benefit compared to bone grafting is that it lowers infection, implant loosening, and scapular notching risks.^32^

Metallic baseplate augmentation can help better manage glenoid bone deficiency, preserve healthy bone, and minimize reaming.^38^ At the ≥ 2-year follow-up of 35 patients after AGB use with rTSA, Wright et al^60^ confirmed the safety and efficacy of posterior and superior AGB. In a study of 139 patients with 1-3 year post-rTSA follow-up, Michael et al^38^ reported an 8% complication rate for those with posterior AGB but no glenoid baseplate failures. Among a patient group with 22 superior, 50 posterior, and 67 postero-superior AGB, Michael et al^38^ reported that each group had improved shoulder function with reduced pain. An AGB was believed to improve deltoid muscle function by lateralizing the glenohumeral center of rotation, helping prevent injurious torque or shear forces.^3^ In a retrospective chart review of 37 patients with 23.3 ± 6.3 month follow-up, Bauer et al^5^ failed to identify glenoid baseplate loosening, hardware failure, radiolucency, or scapular notching with AGB use. In a study of 520 patients post-rTSA, Colasanti et al^11^ reported that compared to glenoid bone grafting, AGB use decreased total surgical time by 50%, reduced intra-operative blood loss nearly 33%, reduced scapular notching by 3 times, reduced other total adverse events by 6 times, and reduced rTSA revision rate by 8 times.

Although glenoid bone grafting in rTSA can improve shoulder mobility, if graft resorption occurs, early baseplate loosening and poor outcomes are more likely.^11^, ^22^, ^23^ Difficult to manage multi-planar glenoid defects may especially benefit from AGB use.^11^ The current study only evaluated “off-the-shelf” metallic AGB. For highly variable, irregular glenoid bone defect patient cases, custom, computerized three-dimensional–printed AGB may better optimize congruency and fit. Using computer modeling, Gutiérrez et al^21^ reported that the combination of a more lateral rTSA glenosphere center of rotation and more inferior alignment increased the impingement-free shoulder abduction motion arc.^21^ In combination with a more varus humeral neck shaft angle (asymmetric polyethylene buildup), smaller, lower profile rTSA implants with variable mini-humeral tray trunnion options and more individualized mini-AGB congruency and fit may have contributed to the improved active shoulder ROM that was observed.^7^, ^34^

The rTSA implant modifications evaluated in the current study were designed to improve shoulder mobility and stability while reducing scapular notching, implant loosening or failure.^22^ Low scapular notching rates were observed for all current study groups with only 1 early implant failure occurring (early glenoid baseplate shifting after central screw breakage). Complex scapular notching issues has led to the development of multiple rTSA surgical approaches and implant designs. The purpose of the current study, however, was not to determine which scapular notching factor was most prevalent but rather to determine which rTSA implant combination within the same surgical approach best reduced this concern. The superior overall active shoulder ROM observed in the group that received both the mini-AGB and mini-humeral tray is encouraging since rehabilitation guidelines were the same for all groups without consideration of potentially improved impingement-free shoulder motion arcs.

### Study limitations

The literature supporting rTSA AGB is growing, but to better confirm patient outcomes and implant survival rates longer follow-up studies are needed with more rigorous study designs. The current study presents important early-to-mid-term information. Future studies, however, should randomly assign a larger number of subjects with similar demographics and diagnoses to equally distributed rTSA implant component comparison groups. Although study surgeons attempted to assign patients to study groups based on perceived best individual needs, group 1 subject data using the implant system available at the time were collected prior to group 2 and group 3 implant development. Study surgeons were experienced with the surgical system, familiar with implant design characteristics, used the same surgical approach, and had the same treatment philosophy. The addition of radiograph and CT scan image review by an independent radiologist would have helped better eliminate potential diagnosis confirmation, scapular notching grade, and complication status bias. Propensity matched control group (eg, age, sex, comorbidities, activity/sports level, performance expectations) inclusion may also help better delineate population-specific rTSA benefits. There is no consensus, however, about which covariates might generate the greatest post-rTSA patient outcome impact.^3^ Current study subject groups had similar age, sex distribution, shoulder symptom duration, body mass index, and alcohol or tobacco use frequency. They also had comparable primary pre-rTSA diagnoses, quality of life (EQ-5D-5 L), ASES shoulder, VAS pain, and instability score improvements across the 5-year post-rTSA study period. The large number of implant design, size, and alignment variables, however, may have generated several additional degrees of freedom that were not effectively controlled. Although the center of rTSA rotation was likely lateralized, the magnitude of this was not directly measured. Also, group 1 subjects also had lower pre-rTSA active shoulder ROM than the other groups. Although these initial differences largely recovered by 6 months or 1 year post-rTSA and non-parametric statistical analysis was less influenced by data outliers across all follow-up periods, future studies should better control this during initial group assignment. Multiple statistical comparisons were used without *P* value corrections. Dividing the alpha value by the total number of statistical comparisons performed, however, as in Bonferroni-type corrections would likely have increased type II errors by making adjusted *P* value significance thresholds too strict, potentially discarding important discoveries in the process.^17^, ^41^, ^47^ The subject group that received lower profile rTSA implants also had more subjects that underwent subscapularis tendon repair. This may have further improved active surgical shoulder ROM, particularly internal rotation. The greater active shoulder flexion, external rotation at 90°, and internal rotation observed in group 3 most likely occurred from the combination of lower profile rTSA components contributing to a larger impingement-fee motion arc and more frequent subscapularis tendon repair. Active shoulder ROM involves a series of synergistic links including trunk or core neuromuscular function, however, which the current study did not measure. Lastly, the addition of objective handheld dynamometry would have better elucidated strength changes over the 5-year data collection period. Despite these limitations, across 5 years post-rTSA subjects receiving the lower profile mini-AGB and mini-humeral tray with 3 trunnion option combination generally had better active shoulder ROM than the other groups. These data should be considered preliminary, and further study with randomized subject assignment of larger subject numbers is recommended.

## Conclusion

Greater active shoulder ROM was observed in subjects that received the lower profile mini-AGB and mini-humeral tray with 3 trunnion options. High implant survival, low scapular notching rates, and PROM improvements were observed for all groups.

## Disclaimers:

Funding: This study was sponsored by Zimmer Biomet.

Conflicts of interest: Ryan Krupp reports that he has received research support from and is a paid consultant for Zimmer Biomet for work related to the subject of this article.

Christopher O'Grady reports that he is a paid consultant for Zimmer Biomet for work related to the subject of this article.

Brian Werner reports that he is a paid consultant for Zimmer Biomet for work related to the subject of this article.

J. Michael Wiater reports that he is a paid consultant for Zimmer Biomet for work related to the subject of this article. Given his role as Editor-in-Chief, J. Michael Wiater had no involvement in the peer-review of this article and has no access to information regarding its peer-review. Full responsibility for the editorial process for this article was delegated to Theodore Blaine.

Thomas Duquin reports that he has received research support from and is a paid consultant for Zimmer Biomet for work related to the subject of this article.

Any additional authors, their immediate families, and any research foundations with which they are affiliated have not received any financial payments or other benefits from any commercial entity related to the subject of this article.

## Ethics declaration

Written informed consent to take part in the study and to publish the article has been obtained from all participants or their legal representatives. The privacy rights of participants have been observed.

This study was performed in compliance with relevant laws, regulatory frameworks and guidelines where the research took place. This study was approved by the Western Institutional Review Board, Inc., Puyallup, WA, USA. (Approval No. CMG2017-70 E)

This research follows the CARE guidelines and the CARE checklist.

The results of this clinical trial and any associated work have been posted in a registry. This clinical trial was registered with number NCT03726554 (Clinical Trials.gov).
